# Vibrational and Electronic Spectroscopy of 2-Cyanoindene Cations

**DOI:** 10.1021/acsearthspacechem.4c00270

**Published:** 2024-12-16

**Authors:** Thomas E. Douglas-Walker, Eleanor K. Ashworth, Mark H. Stockett, Francis C. Daly, Isabelle Chambrier, Vincent J. Esposito, Marius Gerlach, Angel Zheng, Julianna Palotás, Andrew N. Cammidge, Ewen K. Campbell, Sandra Brünken, James N. Bull

**Affiliations:** † School of Chemistry, University of Edinburgh, Joseph Black Building, King’s Buildings, David Brewster Road, Edinburgh EH9 3FJ, U.K.; ‡ School of Chemistry, Norwich Research Park, University of East Anglia, Norwich NR4 7TJ, U.K.; § Department of Physics, Stockholm University, SE-10691 Stockholm, Sweden; ∥ NASA Ames Research Center, Moffett Field, California 94035, United States; ⊥ FELIX Laboratory, Institute for Molecules and Materials, Radboud University, Toernooiveld 7, 6525 ED Nijmegen, The Netherlands

**Keywords:** polycyclic aromatic hydrocarbon, astrochemistry, infrared, action spectroscopy, anharmonicity

## Abstract

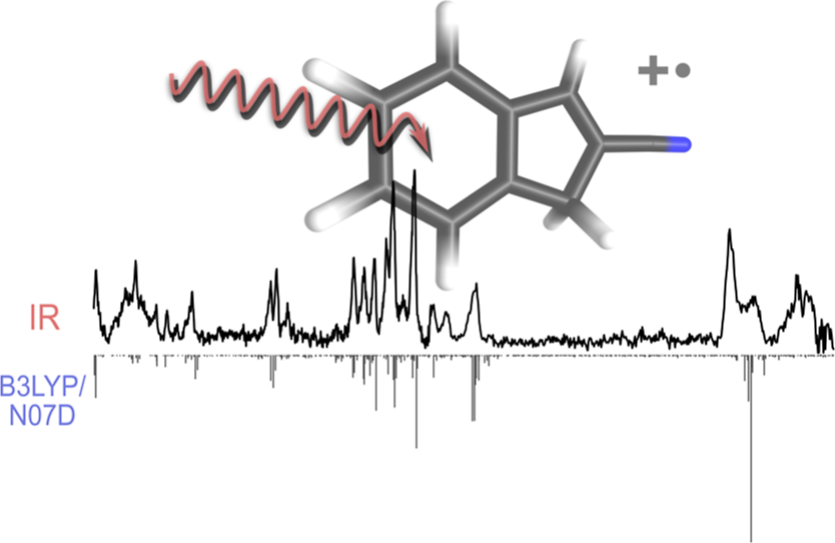

2-Cyanoindene is one of the few specific aromatic or polycyclic aromatic hydrocarbon (PAH) molecules positively identified in Taurus molecular cloud-1 (TMC-1), a cold, dense molecular cloud that is considered the nearest star-forming region to Earth. We report cryogenic mid-infrared (550–3200 cm^–1^) and visible (16,500–20,000 cm^–1^, over the *D*_2_ ← *D*_0_ electronic transition) spectra of 2-cyanoindene radical cations (2CNI^+^), measured using messenger tagging (He and Ne) photodissociation spectroscopy. The infrared spectra reveal the prominence of anharmonic couplings, particularly over the fingerprint region. There is a strong CN-stretching mode at 2177 ± 1 cm^–1^ (4.593 μm), which may contribute to a broad plateau of CN-stretching modes across astronomical aromatic infrared band spectra. However, the activity of this mode is suppressed in the dehydrogenated (closed shell) cation, [2CNI-H]^+^. The IR spectral frequencies are modeled by anharmonic calculations at the B3LYP/N07D level of theory that include resonance polyad matrices, demonstrating that the CN-stretch mode remains challenging to describe with theory. The *D*_2_ ← *D*_0_ electronic transition of 2CNI^+^, which is origin dominated, occurs at 16,549 ± 5 cm^–1^ in vacuum (6041.8 Å in air). There are no correspondences with reported diffuse interstellar bands.

## Introduction

Infrared (IR) observations from the Spitzer and James Webb space telescopes, combined with astrochemical modeling, suggests that 10–25% of galactic carbon exists as polycyclic aromatic hydrocarbons (PAHs).^1−4^ PAHs are thought to dominate the mid-IR emission from a variety of astrochemical environments, leading to the so-called aromatic infrared bands (AIBs).^5,6^ The most prominent AIBs, which are situated on broad emission plateaus with other weaker bands, are found at 3030 cm^–1^ (3.3 μm, C–H stretch), 1612 cm^–1^ (6.2 μm, C–C stretch), 1299 cm^–1^ (7.7 μm, C–C stretch), 1163 cm^–1^ (8.6 μm, C–H in-plane bending), and 893 cm^–1^ (11.6 μm, C–H out-of-plane bending),^1^ and are presumed to arise from IR emission from PAHs following inelastic collisional activation (energy transfer) with other particles or stellar winds, or after absorption of a visible or UV photon and internal conversion to the ground electronic state.^7,8^ Interestingly, the AIBs emitted from various astrochemical environments can be categorized into classes based on patterns of AIB frequencies and intensities.^9−11^ While there is good agreement between the AIBs in a given class, there are distinct differences between each AIB class. This consistency of AIB spectra for a given class suggests that there is either a common set of PAHs responsible for IR emissions, or there is such a diverse array of contributing PAHs (such as functional group substituted forms) that changes in the distribution has little effect on the AIB spectrum.^8,12^ Laboratory data on a variety of known and likely astro-PAHs is desirable to inform on these interpretations.

To date, several specific PAHs (1-/2-cyanonaphthalene,^13^ indene,^14,15^ 2-cyanoindene,^16^ 1-/5-cyanoacenaphthylene,^17^ and cyanopyrene^18,19^) have been identified in Taurus molecular cloud-1 (TMC-1) through observing their rotational lines via radioastronomy. Single-ring aromatic molecules including benzonitrile, cyanocyclopentadiene, and ethynylbenzene have similarly been observed in TMC-1.^20−22^ Although TMC-1 is classified as a cold, dark molecular cloud where molecules are shielded from most high energy radiation (aside from some cosmic rays),^23^ the edges or boundaries of such clouds may have photodissociation region (PDR) transition zones where far-UV photons (6–13.6 eV)^24−26^ emitted from nearby stars create warm regions of gas and dust. Within these edges, PAHs are thought to control gas temperature through the photoelectric effect and radiation penetration field gradients;^27−30^ consequently, PAHs influence the local charge balance.^31,32^ Photoionization and dissociation modeling studies have surmised that small PAHs (e.g., less than 50 atoms) should not survive in UV radiation fields,^33,34^ although those models assumed cooling only through IR emission.

Characterizing the spectroscopy and excited-state dynamics of PAHs likely to exist in space is critical for understanding the interstellar carbon inventory and lifecycle.^1,35^ The first step in the destruction of PAHs is usually ionization, whether through collisions with cations in cloud cores or molecules in stellar winds, direct photoionization in the PDR, or through cosmic rays.^23^ Identifying the observable signatures of cyano-PAH cations, as well as their primary fragmentation products, could be useful in tracing the evolution of cyano-PAHs in space. One of the current PAH conundrums is that the observed abundance of 2-cyanoindene, as well as 1-cyanonaphthalene (1CNN), 2-cyanonaphthalene and indene, in TMC-1 is several orders of magnitude higher than astrochemical models predicted.^13,16^ This discrepancy indicates that the PAH formation and/or stabilization mechanisms assumed in the models are underestimated. For 1CNN, several of the current authors used cryogenic ion storage ring experiments on the cation to demonstrate that a critical mechanism active in the radiative cooling dynamics is recurrent fluorescence,^36,37^ corresponding to electronic fluorescence from thermally populated excited states.^38^ Studies on other small PAH cations^39−42^ (including naphthalene, azulene, and the closed-shell indenyl cation) have demonstrated that recurrent fluorescence similarly occurs in conjunction with IR emission, and is likely a common property of PAHs considering the similarity of their electronic structures. The efficient radiative cooling of the cations, which serves to prevent their dissociation/destruction pathways, combined with their known presence in TMC-1, suggests that the cations could exist in lower luminosity PDRs.^43^ Continued identification of PAHs in space and understanding of their resilience, evolution, and lifecycles requires reliable measurements of spectroscopic properties, including IR and electronic spectra.^44^

**Figure 1:**
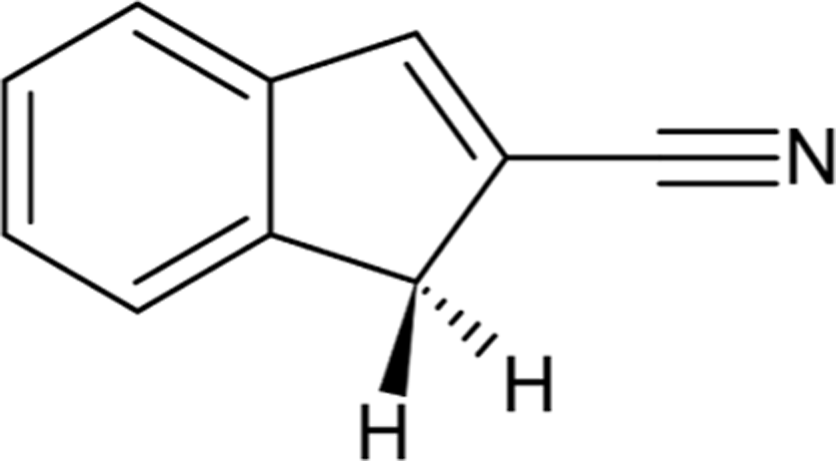
Illustration of the 2-cyanoindene molecule. The radical cation is denoted as 2CNI^+^, with the positive charge delocalized over the molecule. Both neutral and radical cation have an equilibrium geometry with C_s_ point-group symmetry.

Here, we report a simple laboratory synthesis of 2-cyanoindene (not available commercially), hereafter denoted 2CNI (Figure 1), and have used cryogenic messenger tagging spectroscopy in two separate ion trap experiments to record mid-IR vibrational spectra and electronic spectra over the first bright transition (*D*_2_ ← *D*_0_) of the gas-phase radical cation, 2CNI^+^. We also report a preliminary mid-IR spectrum of the closed-shell dehydrogenated form, [2CNI-H]^+^. Messenger-tagging spectroscopy, which is often called infrared predissociation (IRPD) spectroscopy when used with IR radiation, relies on the resonant absorption of radiation by the tagged cation (e.g., 2CNI^+^-Rg, Rg = He, Ne) dislodging the tag atom to generate the untagged cation (e.g., 2CNI^+^). Because the rare gas (Rg) tag atom is only weakly bound (e.g., a few wavenumbers for He and ≈100 cm^–1^ for Ne) and does not participate in chemical bonding, the photodissociation spectra closely resemble absorption spectra for the untagged cation. For 2CNI^+^, we show that the mid-IR spectrum is dominated by the CN stretch at 2177 ± 1 cm^–1^, although dehydrogenation (i.e., [2CNI-H]^+^) strongly suppresses the activity of this mode. In accord with recent modeling of neutral cyano-PAHs,^45,46^ the mid-IR spectra show significant contributions from anharmonic couplings and combination bands. The *D*_2_ ← *D*_0_ electronic spectrum of 2CNI^+^, with an origin transition at 16,544 ± 5 cm^–1^ in vacuum, has a spectral profile similar to the indene and 1CNN^+^, and does not correspond to any known diffuse interstellar bands.

## Methods

### Synthesis

2-Cyanoindene^47^ was synthesized by regiospecific cyanation of 2-bromoindene following the general conditions for similar cyanations described in ref (48). 2-bromoindene, potassium ferrocyanide, copper iodide catalyst, and 1-butylimidazole as ligand were stirred in refluxing *p*-xylene and the reaction monitored by thin-layer chromatography. After 6 h the reaction was worked up by filtering through Celite (EtOAc) followed by washing with brine, and the crude product purified by column chromatography (silica gel, eluent petroleum ether/EtOAc 10:1) to yield the product as a pale yellow crystalline solid (41%). MP. 41.9 °C. The identity and purity of the 2-cyanoindene product was confirmed by ^1^H NMR and ^13^C NMR spectroscopy (see Supporting Information). No unexpected or unusually high safety hazards were encountered.

### IR Spectroscopy at FELIX

IR spectra were recorded using the FELion 22-pole cryogenic ion trap apparatus,^49^ stationed at the free-electron laser for infrared eXperiments (FELIX) laboratory.^50^ Briefly, 2CNI powder was sublimed at room temperature and introduced into the FELion instrument using a variable leak valve. 2CNI^+^ was generated through electron impact ionization (electron energy 23 eV with fwhm of ≈2 eV). Cations of the desired mass (*m*/*z* = 141 for 2CNI^+^ or *m*/*z* = 140 for [2CNI-H]^+^) were selected by the first quadrupole mass filter after a ≈100 ms duration extraction pulse, and transmitted into a 22-pole ion trap that was maintained at a temperature of *T* ≈ 6 K, which was monitored using a silicon diode. Specifications and dimensions of the ion trap are given in ref (51). Trapped ions were cooled through collisions of the ions with a 3:1 mixture of He:Ne admitted into the trap using a piezoelectric pulsed valve (≈80 ms duration pulses), with the collisions resulting in ≈10% of the stored ions forming ion-Ne complexes. The contents of the trap were exposed to FEL-2 radiation (550–2400 cm^–1^, in 1 cm^–1^ increments) from the FELIX laboratory, which had a repetition rate of 10 Hz between macropulses, a typical energy of 10–35 mJ pulse^–1^ at the trap center with a beam waist of ≈2 mm over the length of the trap (40 mm), and line widths of fwhm ≈0.5–1% of the center wavenumber. The beam fluence is thus 320–1100 mJ cm^–2^ pulse^–1^. Experience has shown that the loose focusing conditions allows the FELIX beam to intersect with the entire contents of the trap by the motion of the ions within the trapping field. The radiation beam path between the beamline and instrument was purged to minimize atmospheric contaminants. Resonant excitation of a vibrational mode resulted in dissociation of the ion–Ne complex. The extent of photodepletion of the complex relative to the total number of ions without laser irradiation (by passing the ion trap contents through a second quadrupole mass filter and quantifying with a Daly detector) was measured as a function of wavenumber. Because the ion cloud in the 22-pole ion trap is relatively large, the interaction of 26 or so macropulses with the trapped ions was required to achieve a sufficient photodepletion signal. The FEL-2 wavenumber was calibrated using an IR grating spectrum analyzer. The photodepletion signal at a given wavenumber was normalized to the FEL pulse energy (*E*) and number of shots (*N*) to determine the relative cross section according to
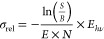
1where *S* is the observed ion count, *B* is the baseline ion count, and *E*_hν_ is the photon energy. The final spectra are the average of three or four repeat acquisitions.

### IR and Electronic Spectroscopy in Edinburgh

IR and electronic spectroscopy measurements at the University of Edinburgh were carried out using a cryogenic ion trapping apparatus described previously.^52−54^ 2CNI^+^ was generated through 30 eV electron-impact ionization of the neutral sample that was introduced into a high-vacuum chamber using a variable leak valve. The target cations were then mass-selected using a quadrupole mass filter and were passed into linear quadrupole ion trap cooled to *T* ≈ 4 K by a helium compressor. In the trap, stored ions were collisionally cooled with helium buffer-gas (≈10^15^ cm^–3^), causing 20–30% of 2CNI^+^ to form 2CNI^+^-He complexes. After a delay of several hundred microseconds to remove excess helium, the contents of the trap were irradiated with laser light. Photodepletion of 2CNI^+^-He complexes was monitored by extracting the trap contents (1 Hz repetition rate) and passing the ions through a second quadrupole tuned to the *m*/*z* of the complex, which was quantified with a Daly detector. The absolute number of 2CNI^+^-He complexes with (*N*_*i*_) and without (*N*_0_) laser radiation was determined using a mechanical shutter. The same experimental setup was used for both IR and electronic spectroscopy measurements.

The IR measurements used an OPO/OPA system (LaserVision, line width ≈0.8 cm^–1^, 4 pulses per trapping cycle), while the electronic spectroscopy measurements used a different OPO/OPA (EKSPLA NT-342B-SH, line width ≈5 cm^–1^, 2 pulses per trapping cycle) or dye laser (Sirah, line width ≈0.05 cm^–1^, 2 pulses per trapping cycle, Rhodamine 610 at 0.2 g L^–1^ in ethanol). The photodepletion signals were corrected for variations in laser fluence as a function of wavenumber (calibrated using a HighFinesse WS6 wavemeter) based on a power curve measured just before the spectral acquisitions. The measured photodepletion signal, which was corrected for the number of background ions (*N*_B_), is given in terms of a cross-section by
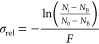
2where *F* is the relative OPO/OPA fluence. Photodepletion at a given wavenumber was averaged over 10–20 trapping cycles, and each spectrum was averaged over 3–5 scans. For electronic spectroscopy measurements, the ion cloud was exposed to radiation with a beam intensity of a few mJ cm^–2^ pulse^–1^. For IR spectroscopy measurements, the ion cloud was irradiated with beam intensities on the order of tens of mJ cm^–2^ pulse^–1^ by focusing the beam into the ion trap (∼1 mm beam diameter) with either a CaF_2_ or ZnSe lens. During measurements, the photodepletion of 2CNI^+^-He complexes did not exceed 40%.

Two-color experiments on the origin transition of bare 2CNI^+^ (*m*/*z* = 141) were carried out by monitoring photoproduction of the *m*/*z* = 114–115 mass channel, which corresponds to loss of HCN/HNC or C_2_H_2_. For these experiments, the ion cloud was irradiated by the EKSLPA OPO/OPA at a fixed wavelength of 500.0 nm, while the Sirah dye laser scanned over the wavenumber range of interest. The two-color measurements were performed at higher trap temperatures (*T* ≈ 8 K) to remove any He-tagged complexes.

### Theoretical

#### Electronic Structure Calculations

2CNI^+^ geometries and anharmonic vibrational frequencies (VPT2 method as implemented in Gaussian 16.B01)^55^ were determined using the cc-pVTZ basis set^56^ with the following density functionals: B3LYP,^57^ CAM-B3LYP,^58^ LC-ωHPBE,^59^ and ωB97X-D.^60^ In addition to bare 2CNI^+^, geometries of Ne- and He-tagged forms were optimized, and anharmonic frequencies computed, at the ωB97X-D/cc-pVTZ level of theory, with the tag atom in two different positions (see Supporting Information). From these geometries, the lowest energy Ne-tagged form was reoptimized with counterpoise corrections and the anharmonic frequencies determined.^61^ Counterpoise corrections were not included for He-tagged species. The optimized structures are summarized in the Supporting Information.

The electronic absorption spectrum of 2CNI^+^ was modeled at the ωB97X-D/cc-pVTZ level of theory using the Franck–Condon-Herzberg–Teller framework as implemented in Gaussian 16.^60,62^

#### Simulation of IR Spectra

Many density functional theory methods fail to reliably reproduce anharmonic frequencies within the VPT2 framework; thus, the IR spectra were also simulated using a more reliable strategy.^45,63^ First, the optimized geometry, normal modes, and harmonic frequencies of 2CNI^+^ and [2CNI-H]^+^ were computed at the B3LYP/N07D level of theory^57,64^ in Gaussian 16.^55^ The N07D basis set, which is based on the 6-31G(d) basis set, was augmented with additional diffuse and polarization functions,^65^ which have been shown to improve the anharmonic computations of large aromatic systems, including PAHs.^66−68^ The computations were performed with very-tight optimization criteria and a custom integration grid consisting of 200 radial shells and 974 angular points per shell.^69^ The quadratic, cubic, and quartic normal force constants (quartic force field, QFF) were then computed at the B3LYP/N07D level of theory via small displacements of atoms along predefined normal mode coordinates. The QFF, which is a truncated fourth-order Taylor series expansion of the potential surrounding the equilibrium geometry, was computed in normal mode coordinates and transformed into Cartesian coordinates via a linear transformation.^70^ Semidiagonal quartic terms (default in Gaussian 16) are included.

After computing the QFF, second-order vibrational perturbation theory (VPT2)^71−75^ was used to compute the anharmonic vibrational spectrum using a modified version of SPECTRO.^76^ The VPT2 method implemented in SPECTRO utilizes a resonance polyad matrix approach.^77,78^ When two vibrational states of the same symmetry are close in frequency, they create a near-singularity in the conventional VPT2 equation. In the present approach, the interacting states are removed from the VPT2 and are included in resonance polyad matrices based on symmetry. This matrix allows for the treatment of resonance effects, while also accounting for states that simultaneously participate in multiple resonance interactions, termed resonance chaining. Additionally, the resonance polyads treat the redistribution of intensity between coupled states by using the eigenvectors of the diagonalized matrix. The maximum frequency separation for a resonance in the polyad treatment is set to 200 cm^–1^.^69^ Vibrational modes with frequencies below 300 cm^–1^ are excluded from the VPT2 treatment due to known issues in the accurate description of their potential energy surfaces.^79−81^ We hereafter refer to this anharmonic computation as the B3LYP/N07D level of theory (Figure 1).

## Results and Discussion

### IR Spectroscopy of 2CNI^+^-Rg (Rg = Ne, He)

**Figure 2:**
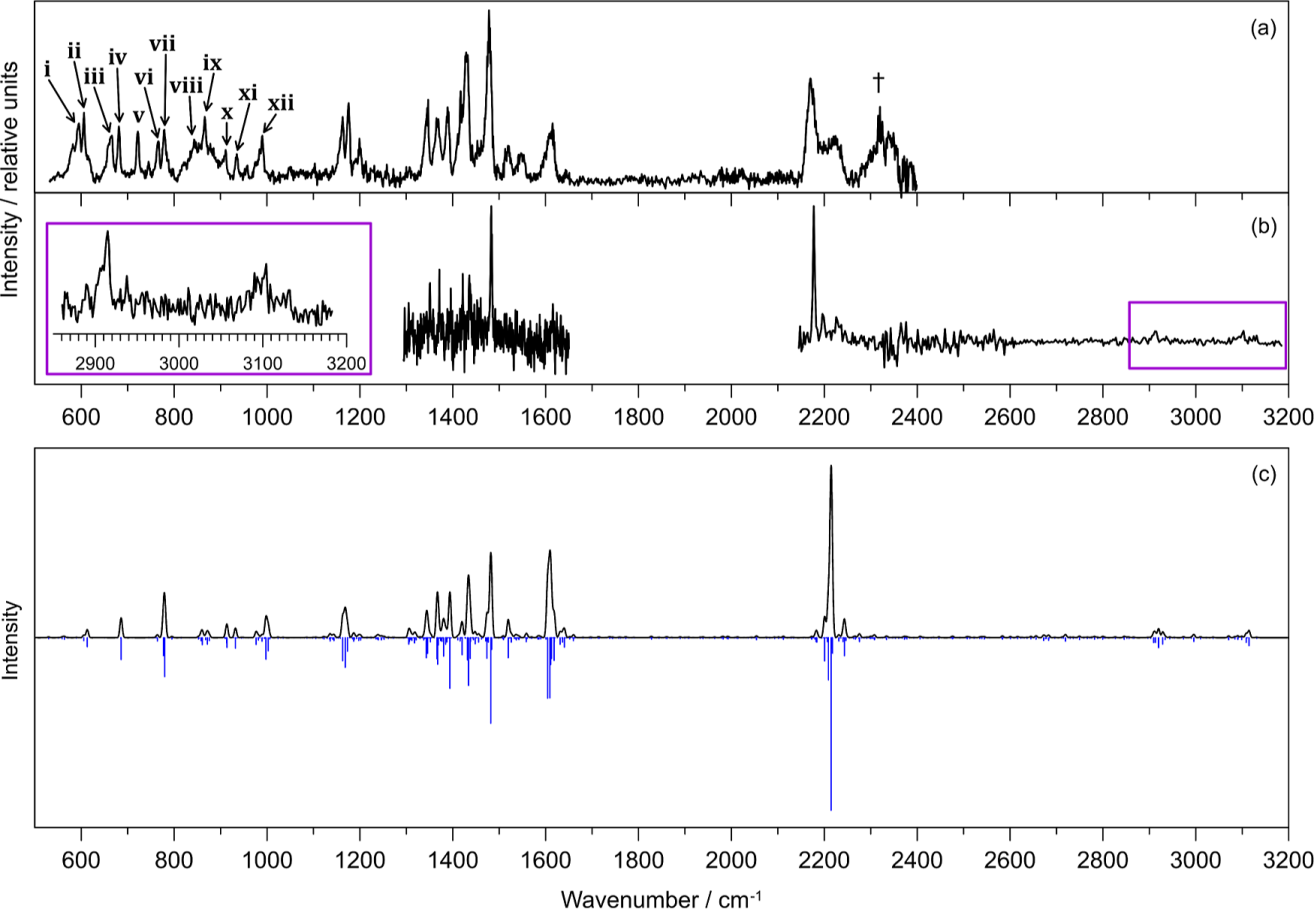
IR spectroscopy of 2CNI^+^-Rg (Rg = He, Ne): (a) 2CNI^+^-Ne photodissociation spectrum recorded at FELIX. Peaks situated below 1000 cm^–1^ are labeled (i) to (xii) to correlate with values in Table 1. The region above 2280 cm^–1^ denoted by † was recorded only once due to changes in accelerator energy over the FELIX beamtime shift allocation and may not be reliable. (b) 2CNI^+^-He photodissociation spectrum recorded in Edinburgh (the inset shows an expansion of the C–H stretch region). Because the beam path was not flushed for atmospheric contaminants, there is higher noise level in the He-tagged spectrum over the CO_2_ asymmetric stretch region (2230–2430 cm^–1^) where the fluence from the OPO dropped. (c) B3LYP/N07D anharmonic calculation. In (c), the black spectrum assumes the stick spectra (blue) convoluted with 5 cm^–1^ fwhm Gaussian functions.

IR spectra recorded for 2CNI^+^-Rg (Rg = Ne, He) are shown in Figure 2. The 2CNI^+^-Ne spectrum (Figure 2a), which covers the 500–2300 cm^–1^, is somewhat congested over the 550–1650 cm^–1^ range, and has CN stretch features at 1900–2200 cm^–1^. While the peak positions (wavenumbers) in this spectrum are reliable, the relative intensities of weaker bands compared with more intense bands may be exaggerated because some of the stronger IR transitions were saturated in order to achieve good signal-to-noise across the whole spectral range. Overall, the spectrum resembles the benzonitrile cation over the fingerprint region.^82,83^ The 2CNI^+^-He spectrum (Figure 2b), covers the 1300–1650 and 2150–3200 cm^–1^ ranges (limited by laser optics), and presents substantially narrower peaks due to the bandwidth of the radiation. Most significantly, the 2150–3200 cm^–1^ range clearly identifies the CN stretching mode at 2177 ± 1 cm^–1^ as well as several weaker modes situated at 2915 and 3104 cm^–1^ (shown inset in Figure 2b). Comparing the two data sets shows that the frequencies with the Ne tag are ≈5 cm^–1^ lower than with the He tag. The observed frequencies from both measurements are listed in Table 1.

**Table 1:** Experimental and Calculated IR Frequencies (in cm^–1^) for 2CNI^+^^a^

2CNI^+^-He^b^	2CNI^+^-Ne^c^	calc	assignment	2CNI^+^-He^b^	2CNI^+^-Ne^c^	calc	assignment
	595 (i)^d^/607 (ii)	612.9	ν_36_, ν_39_ + ν_48_		1519	1519.4	ν_10_
	666 (iii)^d^/682 (iv)	685.8	ν_35_		1547	1558.2	ν_23_ + ν_41_
	721 (v)^d^/765 (vi)	763.9	ν_34_			1609.0	ν_26_ + ν_36_, ν_9_, ν_18_ + ν_43_
	778 (vii)	776.9	ν_42_ + ν_43_		1616	1611.0	ν_18_ + ν_43_, ν_9_
		779.7	ν_33_			1612.8	ν_11_ + ν_47_, ν_9_
	844 (viii)^d^/866 (ix)	867.9	ν_35_ + ν_46_, ν_31_, ν_40_ + ν_42_			1618.2	ν_19_ + ν_41_
		871.6	ν_39_ + ν_44_, ν_31_, ν_40_ + ν_42_			1618.5	ν_28_ + ν_35_, ν_19_ + ν_41_, ν_32_ + ν_33_
		875.6	ν_30_, ν_39_ + ν_44_	2177.3	2171	2172.9	ν_17_ + ν_31_, ν_17_ + ν_30_
	911 (x)	913.2	ν_37_ + ν_44_, ν_29_		2227	2182.3	ν_17_ + ν_30_, ν_17_ + ν_31_, ν_13_ + ν_34_
	935 (xi)	932.0	ν_28_, ν_37_ + ν_44_			2184.1	ν_13_ + ν_34_, ν_17_ + ν_30_
	993 (xii)	997.7	ν_26_			2200.8	ν_12_ + ν_34_
	1163	1163.0	ν_21_, ν_20_			2206.7	ν_16_ + ν_31_
	1175	1168.4	ν_20_, ν_21_			2209.0	2ν_24_, ν_8_
		1186.7	ν_34_ + ν_41_			2214.6	ν_8_, 2ν_24_
	1200	1201.5	ν_27_ + ν_45_, ν_19_			2216.3	ν_16_ + ν_30_
1350.3	1344	1342.9	ν_16_, ν_25_ + ν_44_			2217.7	ν_9_ + ν_36_
		1345.9	ν_25_ + ν_44_, ν_16_			2231.3	ν_15_ + ν_31_
1371.2	1368	1365.9	ν_26_ + ν_43_, ν_15_			2240.1	ν_15_ + ν_30_, ν_14_ + ν_30_
		1367.5	ν_15_, ν_26_ + ν_43_			2243.3	ν_23_ + ν_24_, ν_11_ + ν_34_
		1368.8	ν_29_ + ν_40_			2246.6	ν_11_ + ν_34_, ν_23_ + ν_24_
1395.6	1390	1393.8	ν_14_, ν_27_ + ν_42_	2914.9		2909.3	ν_11_ + ν_12_
1421.4	1416	1420.1	ν_13_, ν_26_ + ν_41_			2913.0	ν_9_ + ν_17_, ν_7_
1435.7	1431	1431.5	ν_27_ + ν_40_, ν_12_			2919.9	ν_7_, ν_9_ + ν_17_
		1434.0	ν_12_, ν_27_ + ν_40_, ν_30_ + ν_38_			2928.8	ν_6_
		1437.5	ν_30_ + ν_38_, ν_12_	3104.1		3108.4	ν_2_, ν_1_, ν_4_
1482.9	1479	1481.8	ν_11_, ν_30_ + ν_36_			3114.6	ν_11_ + ν_12_, ν_9_ + ν_11_, ν_2_
		1483.9	ν_30_ + ν_36_, ν_31_ + ν_36_, ν_11_				

a Calculated values are at the B3LYP/N07D level of theory for the bare cation (see methods section); intensities are given in the Supporting Information (see zip file). The mean absolute error (MAE) between theory for the bare cations and messenger-tagged experimental data is 4.3 cm^–1^ (excluding CN stretch). For a graphical comparison of the correlation between experiment and computations, see the Supporting Information. The 2CNI^+^-Ne binding energy is ≈100 cm^–1^ from counterpoise-corrected CCSD(T)/cc-pVTZ calculations (see Supporting Information).

b Uncertainties are ±1 cm^–1^.

c Uncertainties are ±2 cm^–1^ based on a spectrum analyzer calibration.

d No frequency with substantial intensity is found in calculations and was excluded from MAE determinations.

Our best simulation of the IR spectrum (B3LYP/N07D level of theory) for untagged 2CNI^+^ is shown in Figure 2c, and is mostly consistent with experimental frequencies over the 1000–3200 cm^–1^ range, allowing for the mode assignments given in Table 1. For wavenumbers below 1000 cm^–1^, the 2CNI^+^-Ne spectrum is substantially more congested than predicted by theory (several peaks denoted by superscript “d” in Table 1 are not predicted by calculations), which may be due to several factors, including the enhancement of apparent weak combination bands in the experiment, or transition splittings associated with several binding modes/sites of the Ne tag atom, e.g. combination modes involving Ne. Consequently, assignments for wavenumbers below 1000 cm^–1^ should be considered tentative. The mean absolute error (MAE) between the B3LYP/N07D frequencies for the bare cation and experiment (with Ne messenger tagging) is 4.3 cm^–1^ (5.1 cm^–1^ including the CN-stretching mode), and is significantly better than the MAE obtained from several other density functionals (all containing some long-range correction): 15.4 cm^–1^ for CAM-B3LYP, 26.1 cm^–1^ for LC-ωHPBE, and 13.5 cm^–1^ for ωB97X-D/cc-pVTZ. B3LYP with the cc-pVTZ basis set gave an MAE of 6.3 cm^–1^. Our MAE values do not include the stretching frequency for the CN group, as this mode has proven particularly challenging for theory (see below). The MAE value for the B3LYP/N07D methodology excluding modes below 1000 cm^–1^, as they were difficult to assign reliably, was almost unchanged at 4.1 cm^–1^. A full summary of MAEs is given in the Supporting Information. In addition to variations in frequency with the density functional, there are substantial differences in the calculated intensities (see Supporting Information). While the 2CNI^+^-Ne spectrum intensities cannot be reliably compared with theory due to saturation, the 2CNI^+^-He spectrum intensities appear in best overall agreement with the B3LYP/N07D calculation. It should be noted that, while B3LYP is fairly criticized for poor performance in systems requiring dispersion interactions and charge–transfer processes (particularly in a time-dependent framework),^84−86^ the parametrization in the B3LYP functional was optimized for main-group bonding parameters, which are those most important for defining vibrational frequencies.

The vibrational mode assignments in Table 1, which includes only the modes that contribute at least 10% to a given mixed vibrational transition, show substantial contributions from combination bands. In accord with calculations on neutral cyano-PAHs using the same B3LYP/N07D methodology,^45,46^ the high intrinsic intensity of the fundamental CN-stretching mode is redistributed to nearby states through anharmonic coupling and consequently leads to a complex spectrum. Furthermore, because there are often many shoulder transitions predicted within a few wavenumbers or less from a measured central transition (i.e., the shoulders are not experimentally resolved), we must assume that several of the calculated transitions can contribute to the observed bands. A tabulation of the calculated fundamental modes is given in the Supporting Information as well as a complete tabulation of the computed spectrum, with intensities, as a zip file.

**Figure 3:**
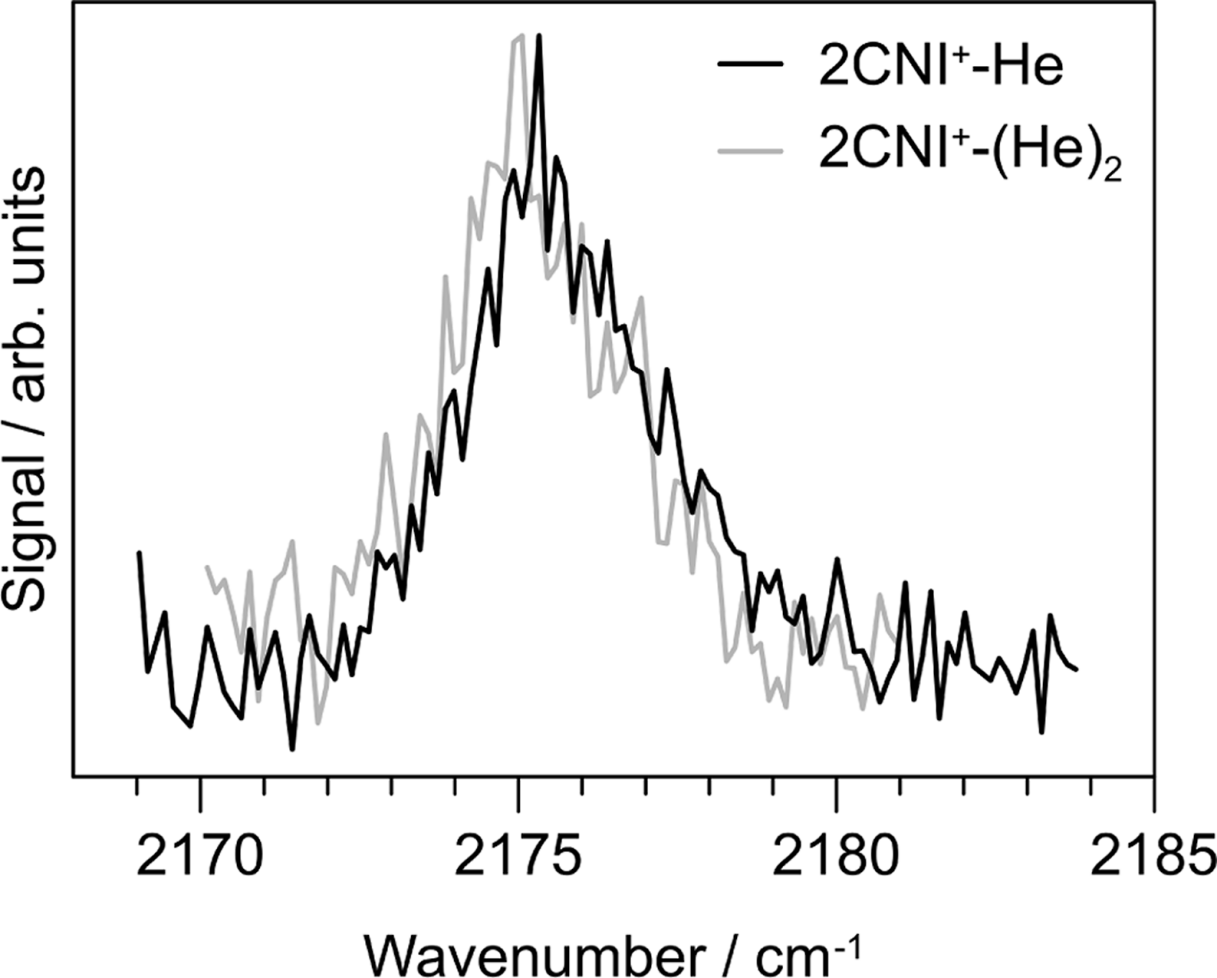
IR spectroscopy of 2CNI^+^-He (black) and 2CNI^+^-He_2_ (gray) over the CN-stretch mode. The line shape is asymmetric, consistent with either contributions due to unresolved transitions from anharmonic coupling (see calculations in Table 1), or the rotational envelope of the vibration.

The most significant feature in the IR spectra is at 2177 ± 1 cm^–1^ (He tag) and is associated with ν_8_ (CN stretching mode). This corresponds to the transition calculated at 2214.6 cm^–1^, which possesses the most CN-stretch character, however, the surrounding modes all include some ν_8_ character. Of all the observed IR bands, there was greatest deviation between theory and experiment for the CN stretch (37 cm^–1^); thus, the MAEs discussed above were calculated with and without this mode included. The other intense IR modes in the 1300–1650 cm^–1^ range are associated with ring stretching and breathing. The CN-stretch mode for 2CNI^+^-He at 2177 ± 1 cm^–1^ (4.593 μm) is slightly higher frequency than that for the Ne-tagged and He-tagged benzonitrile cation at 2120 ± 1 cm^–1^^82,83^ and 2130 ± 1 cm^–1^,^87^ respectively, and lower frequency than He-tagged 1CNN^+^ at 2214 ± 1 cm^–1^.^54^ To ascertain the influence of the He-tag atom on the central transition frequency for 2CNI^+^, we performed smaller-increment wavenumber scans over the CN-stretching band (Figure 3) for 2CNI^+^-He and 2CNI^+^-He_2_, which indicated a 0.3 cm^–1^ red-shift induced by a second helium (similar in magnitude to 1CNN^+^).^54^ Assuming a linear correlation in frequency shift with the number of complexed heliums, the bare 2CNI^+^ transition should be at 2177.6 cm^–1^.

The 2900–3200 cm^–1^ region for 2CNI^+^-He (Figure 2b, inset) shows the C–H stretching region, revealing several weak groups of bands. These weak features are consistent with other studies showing that PAH cations tend to lose almost all of the C–H stretch intensity compared to the neutral molecule.^31,66,88^ The origin of this effect can be traced back to the fact that the positive charge is delocalized over the aromatic ring in PAH cations. Specifically, for the neutral molecule, the C–H bonds are slightly polar with the more electronegative carbon resulting in a partial positive charge on the hydrogen. This causes a substantial change in the dipole moment for the C–H stretching motion. In the case of PAH cations, there is a positive charge localized in the π-system of the ring that effectively reduces the C–H bond polarity. The two positive charges moving away from each other in a C–H stretching motion results in a small dipole change and a lower IR intensity.

Our discussion so far pertains to comparing messenger-tagged IR spectra with calculations for bare (untagged) 2CNI^+^ because anharmonic calculations for weakly bound messenger-tagged species are very difficult due to the shallow potential energy surfaces for binding of the tag atom. While the perturbations introduced from He tagging are usually small at a few wavenumbers or less, perturbations from Ne tagging may be larger.^89−92^ To help inform on the effect of the tag atom, we optimized the geometries of the two lowest energy structures of 2CNI^+^-Rg (Rg = He, Ne) and computed anharmonic vibrational frequencies at the ωB97X-D/cc-pVTZ level of theory. For both tag atoms, the two geometries (see Supporting Information) have relative energies within a few wavenumbers of each other and are expected to coexist in the experiment. For the He complexes, the complex binding energies are only a few wavenumbers, while for the Ne species, the binding energies of the complexes are ≈100 cm^–1^ (CCSD(T)/cc-pVTZ with counterpoise corrections). The calculated anharmonic IR spectra with and without tag atoms show substantial variations in band intensities and frequencies over the 550–1000 cm^–1^ range. Thus, combined with the knowledge from other experiments that IR frequencies can vary by a few to tens of wavenumbers depending on the binding site of “polarizable” tag atoms,^93^^93−95^ we conclude that some of the vibrational congestion observed over the 550–1000 cm^–1^ range for the Ne complexes likely is a consequence of tag atom perturbations and multiple tag atom binding sites.

Emission bands from CN-containing PAHs may contribute to the AIBs through radiative cooling. For example, emission bands from the ionization bar in Orion observed at 4.4 and 4.65 μm, which have been suggested to arise from deuterated PAHs,^96^ are close to the predicted CN-stretch modes for small cationic cyano-PAHs. However, as outlined in the introduction, the AIBs are complex with broad emission plateaus and weak features, and show distinct variation over AIB classes. A recent theoretical study by one of the present authors, using the B3LYP/N07D methodology, has calculated CN-stretch bands in the 4.3–4.5 μm (≈2300–2175 cm^–1^) region for a series of neutral cyano-PAHs (deviating by 60 cm^–1^ for neutral benzonitrile),^45^ potentially contributing to an underlying AIB plateau feature. Thus, the prominence of the CN-stretch band in this region for cationic cyano-PAHs is a useful spectroscopic marker. AIB widths (depends on AIB class) for the most prominent bands are typically 20–40 cm^–1^,^4^ ideally requiring theoretical methods to produce vibrational frequencies with a precision of 10 cm^–1^ or better. Therefore, our MAE value at 4.3 cm^–1^ (excluding CN-stretching) for the carbon backbone is acceptable, although the deviation of the CN-stretch mode (37 cm^–1^) should be addressed in method development. Further measurements on cyano-PAHs spanning a range of charge states are needed considering the extent of anharmonic couplings and the particular difficulty of electronic structure methods in describing CN-stretch mode frequencies.

### IR Spectroscopy of [2CNI-H]^+^-Ne

**Figure 4:**
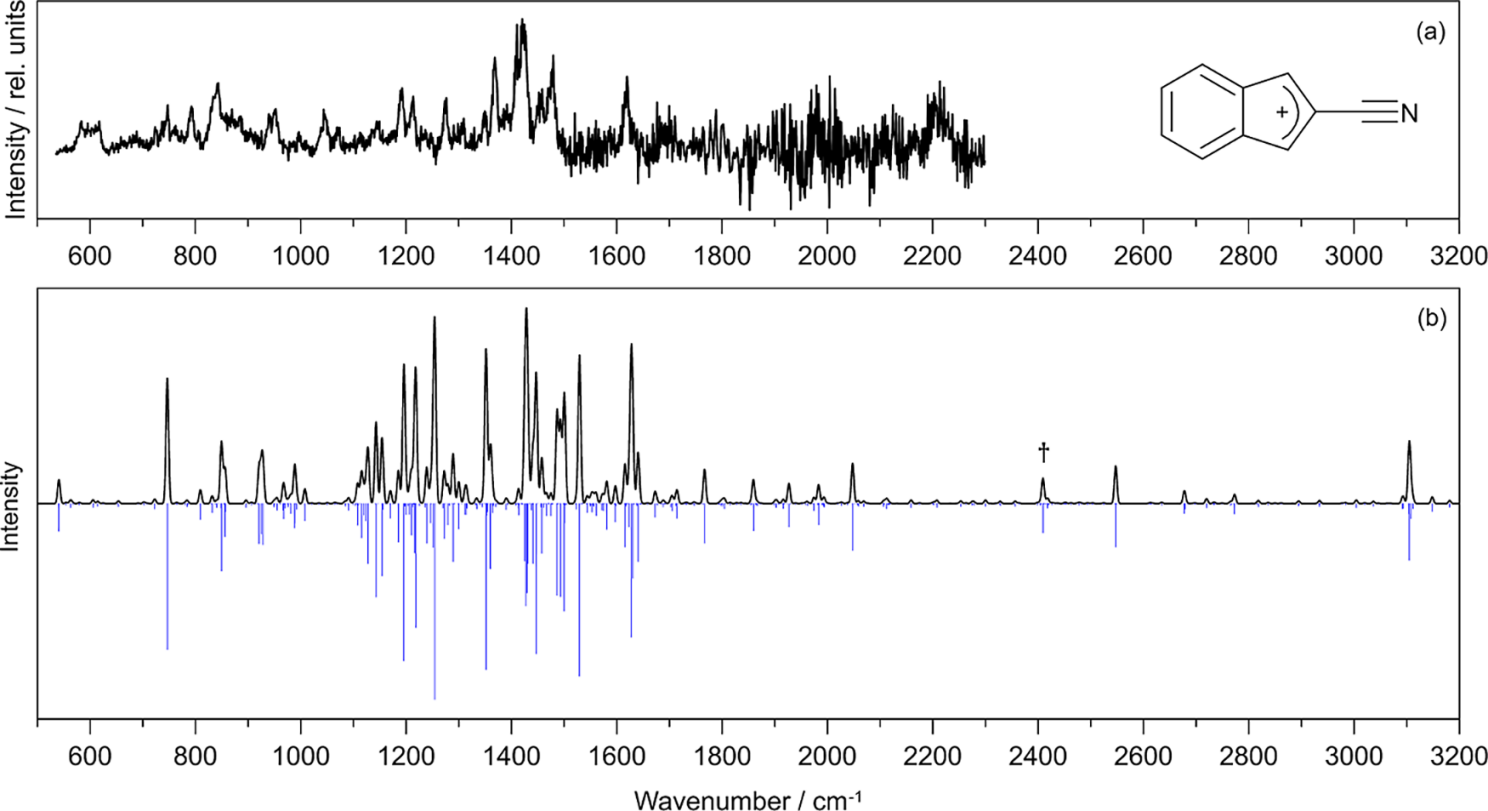
Infrared spectroscopy of [2CNI-H]^+^-Ne: (a) photodissociation spectrum recorded at FELIX. (b) Anharmonic spectrum computed with the B3LYP/N07D methodology. In (b), the black spectrum assumes the stick spectra (blue) convoluted with 5 cm^–1^ fwhm Gaussian functions. The CN-stretching mode is strongly suppressed, and is indicated by † in (b) at ≈2409 cm^–1^.

The IR spectrum for [2CNI-H]^+^-Ne recorded at FELIX is shown in Figure 4a. [2CNI-H]^+^ was the most abundant fragment in the mass spectra using 20–40 eV electron ionization, consistent with it being associated with the lowest bond dissociation energy. The IR spectrum has lower signal-to-noise than that for 2CNI^+^-Ne because the dehydrogenated complex was less efficiently formed in the experiment. The isomer of [2CNI-H]^+^ shown in Figure 4a is the only one expected in the experiment since other possible dehydrogenation site isomers are >2 eV higher in energy. The B3LYP/N07D simulation is shown in Figure 4b. Assuming the simulation framework produced reliable intensities, the CN-stretch mode, which was pronounced for 2CNI^+^ as well as other CN-bearing radical cations,^54,82,83,87^ is much weaker (marked on the simulation in Figure 4b with the † at ≈2409 cm^–1^). In a simple framework, the most active vibrational transitions are those that invoke a substantial change in the dipole moment. Calculated values of the permanent dipole moment |μ| are 4.86 D (2CNI^+^) and 6.34 D ([2CNI-H]^+^), with the CN group principally responsible for the magnitude of the dipole moment. Analysis of electrostatic potential (ESP) contours for 2CNI^+^ and [2CNI-H]^+^ suggests there is an increase in the positive charge on the two C–H bonds adjacent to the CN group and some additional positive charge at the CN-substituted carbon atom for the [2CNI-H]^+^ molecule. This difference in charge distribution could possibly counteract the dipole-moment change from the CN stretch, thus suppressing the activity of the CN-stretch mode. Further experiments and supporting anharmonic calculations on dehydrogenated cyano-PAH cations (and corresponding neutrals), including isomers, are needed to ascertain the significance of this predicted CN-stretch mode suppression, and consequently how this suppression might influence the radiative cooling dynamics.^36,37,41,42^

### Electronic Spectroscopy of 2CNI^+^

**Figure 5:**
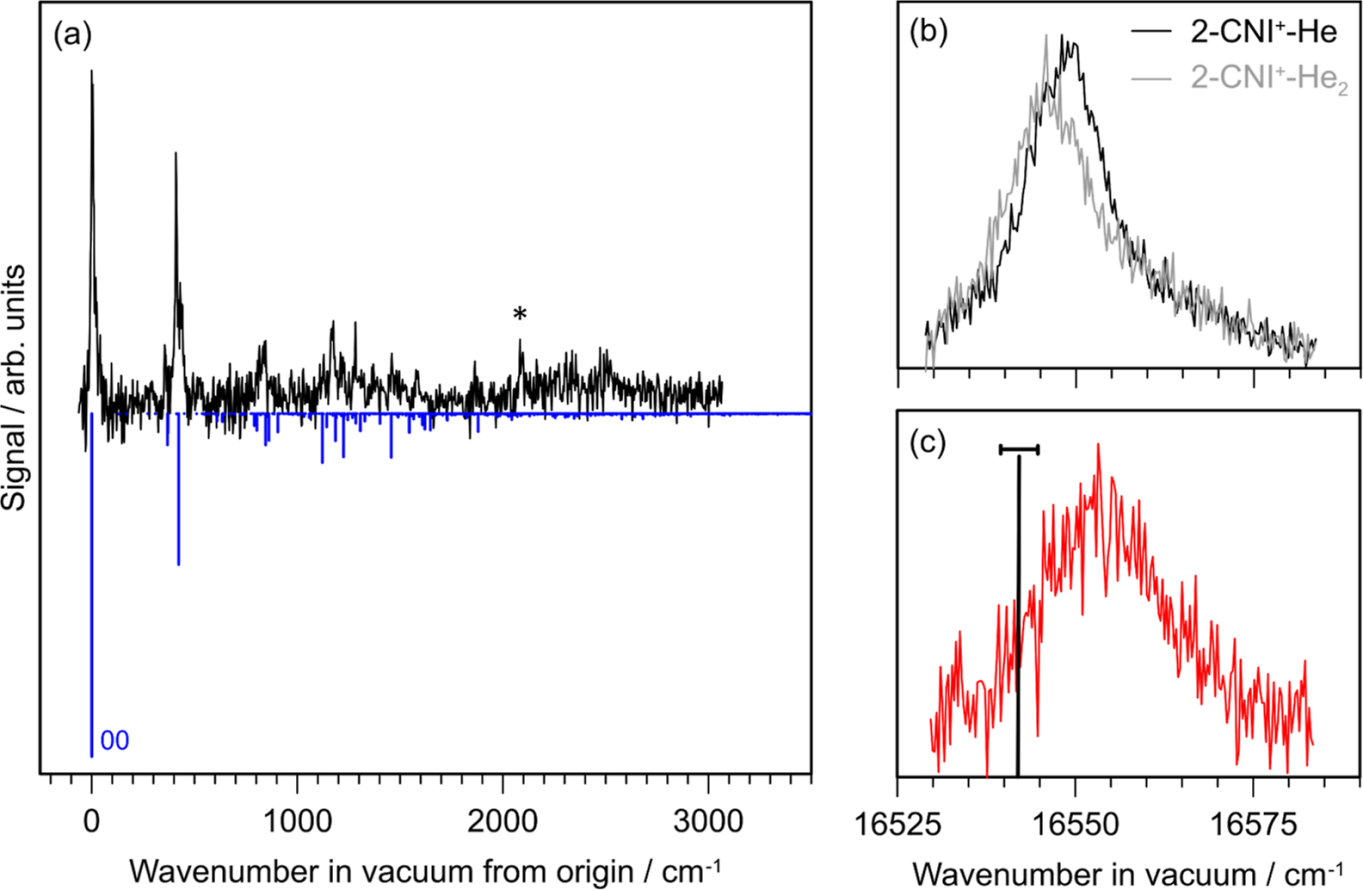
Electronic spectroscopy of 2CNI^+^-He: (a) photodissociation spectrum and Franck–Condon–Herzberg–Teller simulation for the *D*_2_ ← *D*_0_ transition. (b) Band origin measured for 2CNI^+^-He (black) and 2CNI^+^-He_2_ (gray). (c) Two-color measurement of the band origin. The feature denoted with the * in (a) is not reproduced in the simulation, and is either from anharmonic couplings (appears close in frequency to the CN-stretch mode) not included in the simulation or a contaminant species in the experiment at the same *m*/*z*. The vertical bar and uncertainty in (c) indicates the 6045.3 ± 0.8 Å DIB from the reddened B7 la star HD 183143.^98^ The calculated oscillator strength for the *D*_2_ ← *D*_0_ transition is *f* = 0.13. The spectra in this figure, plotted in terms of wavelength in air (Å), are given in the Supporting Information.

The electronic spectrum of 2CNI^+^-He recorded over the *D*_2_ ← *D*_0_ transition is shown in Figure 5a, and is dominated by the origin transition at 16,545 ± 5 cm^–1^ in vacuum (6042.8 Å in air). The spectrum in terms of wavelength in air (Å) is given in the Supporting Information. The electronic spectrum shows clear vibronic structure, which was reproduced in a Franck–Condon–Herzberg–Teller simulation. Herzberg–Teller active modes contribute ≈5% of the total simulated spectral intensity. The band origin with one and two helium tags is shown in Figure 5b, revealing a 3.8 cm^–1^ shift between the two spectra, thus allowing extrapolation of the band origin for the bare ion to 16,549 ± 5 cm^–1^ in vacuum (6041.8 Å in air). A two-color measurement of the origin band for the bare cation, measured by multiphoton fragmentation, is shown in Figure 5c, providing the band maximum at 16,550 ± 5 cm^–1^ in vacuum (6040.5 Å in air), which is within uncertainty of the extrapolated value. The origin band of the bare ion is broader than in the He-tagged data (fwhm = 4.8 Å), attributed to contributions from both one-color and two-color multiphoton fragmentation processes. Thus, the width of the He-tagged band is more likely representative of the 2CNI^+^ origin band at *T* ≈ 10 K. It is interesting to note that the electronic spectrum progression appears similar (but shifted in wavenumber) to those recorded for the 1CNN^+^^53^ and the indene cation,^97^ consistent with the fact that the vibronic structure primarily arises from a common π–π* transition and coupled stretching modes, with little perturbation from the CN group. Calculated natural transition orbitals, shown in the Supporting Information, are consistent with this conclusion.

Interestingly, our origin transition is close to a diffuse interstellar band (DIB) from the reddened B7 la star HD 183143, reported at 16537.3 ± 2.4 cm^–1^ (6045.3 ± 0.8 Å) with a broad fwhm of 78 cm^–1^ (14.2 Å).^98,99^ However, we find no correspondences between the other transitions in Figure 5a and reported DIBs, implying a coincidence. In a more recent DIB survey,^100^ this broad DIB was ascribed to a blend with a second broad DIB at 6037 Å, potentially with additional narrow interstellar features. In many regards, it is not surprising that 2CNI^+^ is not a major DIB carrier considering that (neutral) indene and 1CNN have been observed in TMC-1 through radioastronomy,^13,14^ yet there are no DIB matches with recorded radical cation electronic spectra^53,97,101^ (and DIBs are not observed toward TMC-1). Cationic indene has an origin band with a similar fwhm to 2CNI^+^, whereas the 1CNN^+^ origin transition is substantially broader with fwhm ≈28 Å, meaning that the latter is much more difficult to detect. However, we acknowledge that the lack of correspondence of any DIBs to the *D*_2_ ← *D*_0_ transition in 2CNI^+^ may be a result of low abundance and too low detection efficiencies of astronomical spectrographs.

In a similar vein to the discussion for 1CNN^+^ and hexabenzocoronene cations,^53,102^ we can approximate an upper limit to the astronomical column density of 2CNI^+^ from . By taking the origin band oscillator strength of *f* = 0.015, λ as the measured origin transition wavelength, and the equivalent width *W* as 5 mÅ (from the detection limit of a DIB with fwhm of 4.8 Å),^103^ upper limits to the column density can be estimated as *N* ≈ 1 × 10^12^ cm^–2^ over regions for which DIBs have been observed (not TMC-1, which is a dark molecular cloud). This upper limit of *N* is comparable with those similarly estimated for 1CNN^+^ at ≈10^12^ cm^–2^ (neutral 1CNN has been observed in the same region of TMC-1 as 2CNI), and is an order of magnitude lower than the observed column density for C_60_^+^ in diffuse clouds toward HD 183143 at ≈2 × 10^13^ cm^–2^.

## Conclusions

The gas-phase mid-IR and electronic (*D*_2_ ← *D*_0_) spectroscopy of the 2-cyanoindene radical cation and the mid-IR spectroscopy of the closed-shell dehydrogenated cation have been investigated using cryogenic messenger tagging spectroscopy. The IR spectra reveal the CN stretch mode at 2177 ± 1 cm^–1^ as a major feature in the mid-IR spectra, which would presumably contribute to a broad astronomical marker for cyano-bearing PAHs in space. For 2CNI^+^, we found that several common density functionals, even within the usual VPT2 anharmonic framework, returned band frequencies and intensities in poor agreement with experiment. Computations using B3LYP/N07D second-order vibrational perturbation theory with resonance polyad matrices for the bare cation gave reasonable agreement with experimental frequencies above 1000 cm^–1^, although the CN-stretch mode remains particularly challenging. For frequencies below 1000 cm^–1^, the experimental spectrum is complicated and clear comparisons with theory are difficult. Our theory-experiment comparisons considered only transition frequencies, because intensities are more difficult to reliably measure using difference (photodepletion) action spectroscopy techniques. Ultimately, further measurements of mid-IR spectra for CN-bearing and other hetero-PAHs should be performed in order to establish a larger-scale theory-experiment comparison for the calibration of theoretical methods. Such comparisons are important for small, substituted PAHs so that theoretical methods can be applied with confidence to larger PAHs that are more challenging to study experimentally.

While it is unsurprising that 2CNI^+^ is not a carrier of any observed diffuse interstellar bands, it may be present around the edges of dark molecular clouds where there is a weak UV field. The continued characterization of electronic spectra for likely astro-PAHs is important to gauge limits on PAHs in space and provide critical data required to calibrate astrochemical models.

## Supporting Information Available

The Supporting Information contains details on ^1^H and ^13^C NMR spectra for 2-cyanoindene, summary of the performance of density functional theory methods for anharmonic vibrational frequencies, electronic spectra in terms of wavelength in air (Å), details of tag-atom geometries, illustration of natural transition orbitals for the *D*_2_ ← *D*_0_ electronic transition, listing of the 2CNI^+^ fundamental modes and assignments (PDF)
Anharmonic computational data for 2CNI^+^ and [2CNI-H]^+^ (with the format of “frequency_intensity”) provided as a zip file (ZIP)
