# Supplementary material for: Vibrational and Electronic Spectroscopy of 2-Cyanoindene Cations
Source: ACS Earth Space Chem. 2024 Dec 16;9(1):134–45. doi: 10.1021/acsearthspacechem.4c00270 (PMC11744931; doi:10.1021/acsearthspacechem.4c00270)
Supplement: Supplementary file 1 — sp4c00270_si_001.pdf [file sp4c00270_si_001.pdf]

Supporting Information for 'Vibrational and Electronic Spectroscopy of 2-Cyanoindene Cations'

Thomas E. Douglas-Walker,[1] Eleanor K. Ashworth,[2] Mark H. Stockett,[3] Francis C. Daly,[1] Isabelle Chambrier,[2] Vincent J. Esposito,[4] Marius Gerlach,[5] Angel Zheng,[1] Julianna Palotás,[1] Andrew N. Cammidge,[2] Ewen K. Campbell,[1] Sandra Brünken,[5] and James N. Bull[2]

[1]*School of Chemistry, University of Edinburgh,*
Joseph Black Building, King's Buildings,
David Brewster Road, Edinburgh EH9 3FJ, United Kingdom

[2]*School of Chemistry, Norwich Research Park,*
University of East Anglia, Norwich NR4 7TJ, United Kingdom

[3]*Department of Physics, Stockholm University, SE-10691 Stockholm, Sweden*

[4]*NASA Ames Research Center, Moffett Field, CA 94035, USA*

[5]*Radboud University, FELIX Laboratory,*
Institute for Molecules and Materials,
Toernooiveld 7, 6525 ED Nijmegen, The Netherlands

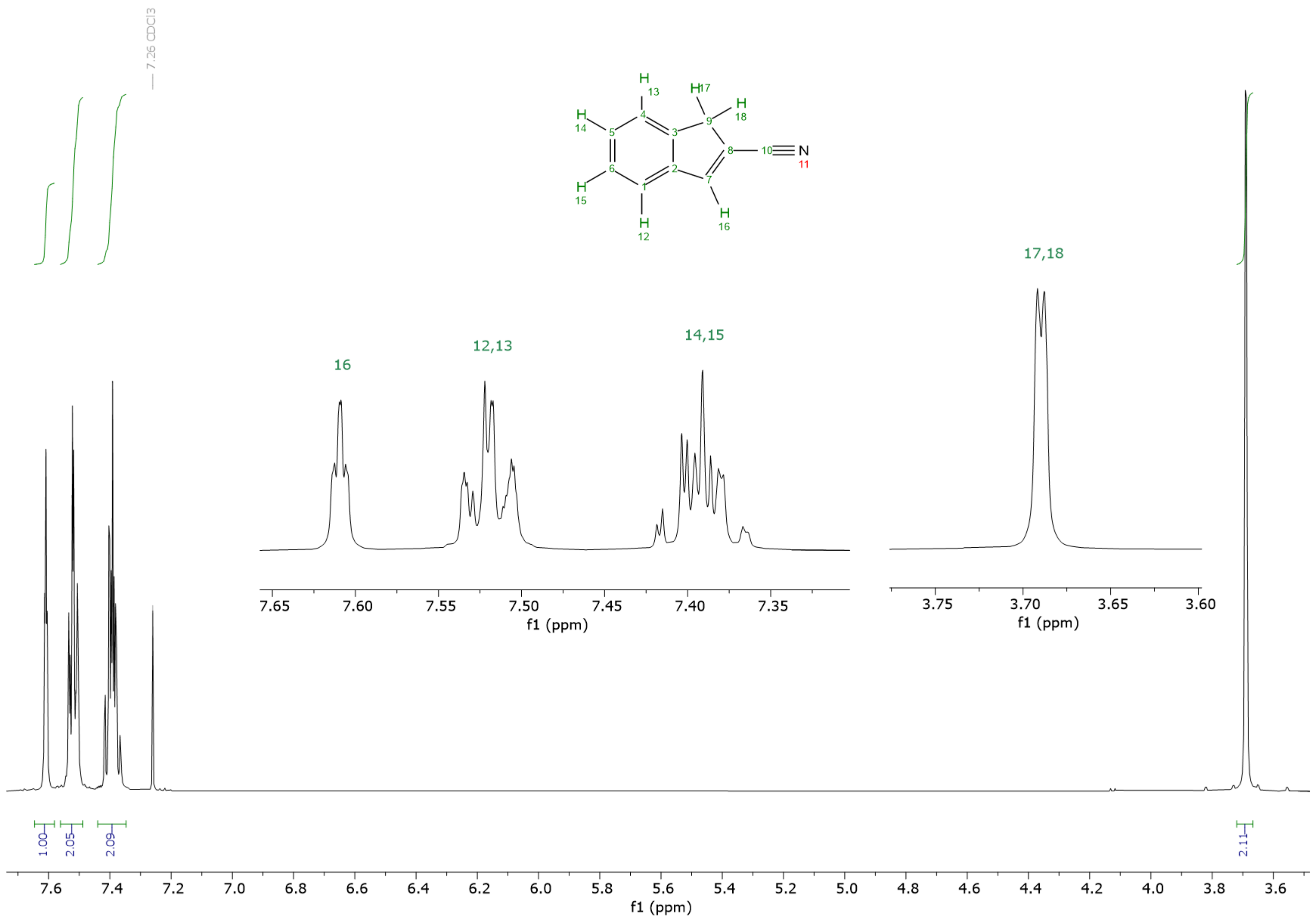

FIG. S1. ^{1}H-NMR spectrum for 2-cyanoindene. The spectrum is consistent with a single structural isomer.

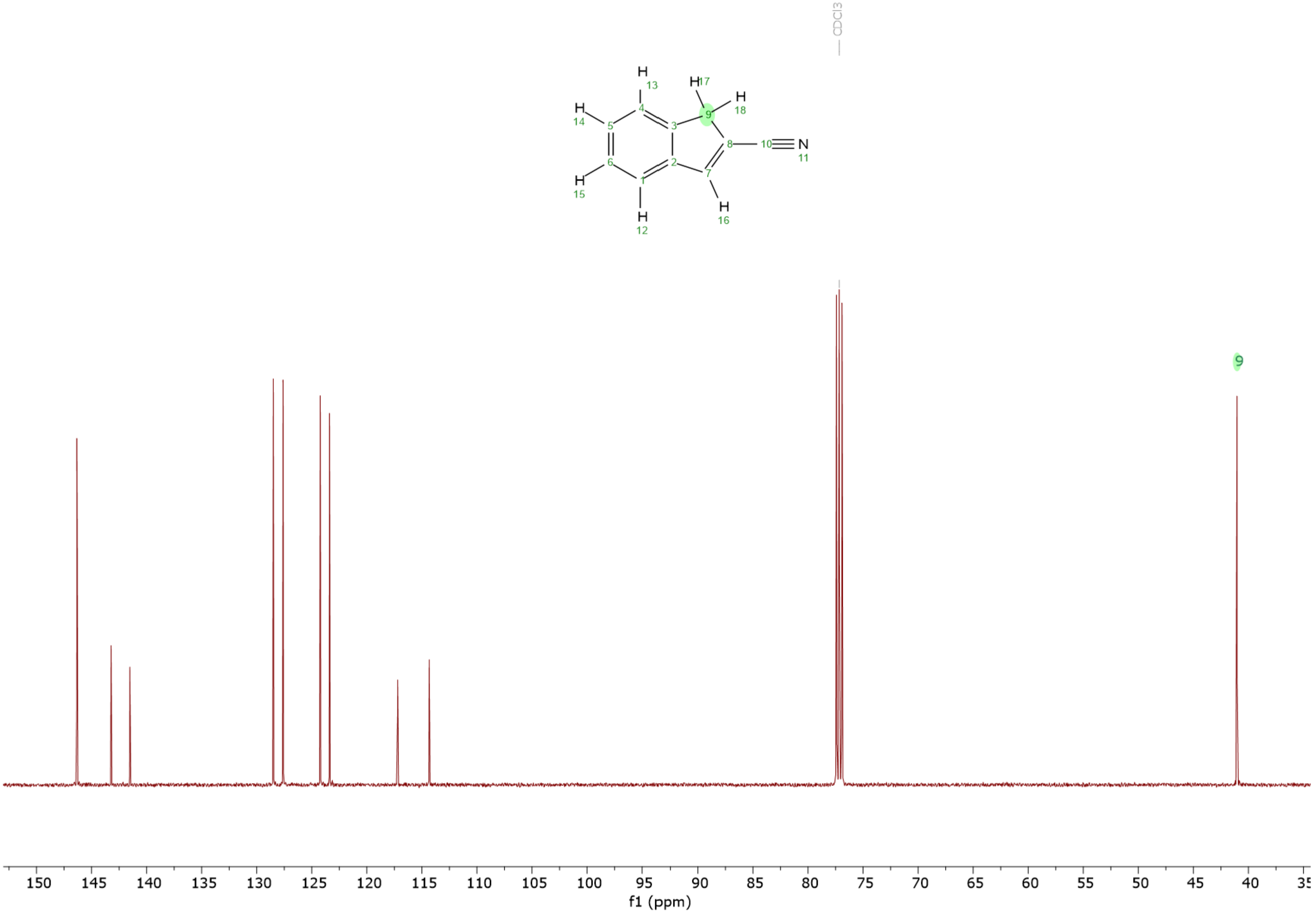

FIG. S2. ^{13}C-NMR spectrum for 2-cyanoindene. The spectrum is consistent with a single structural isomer.

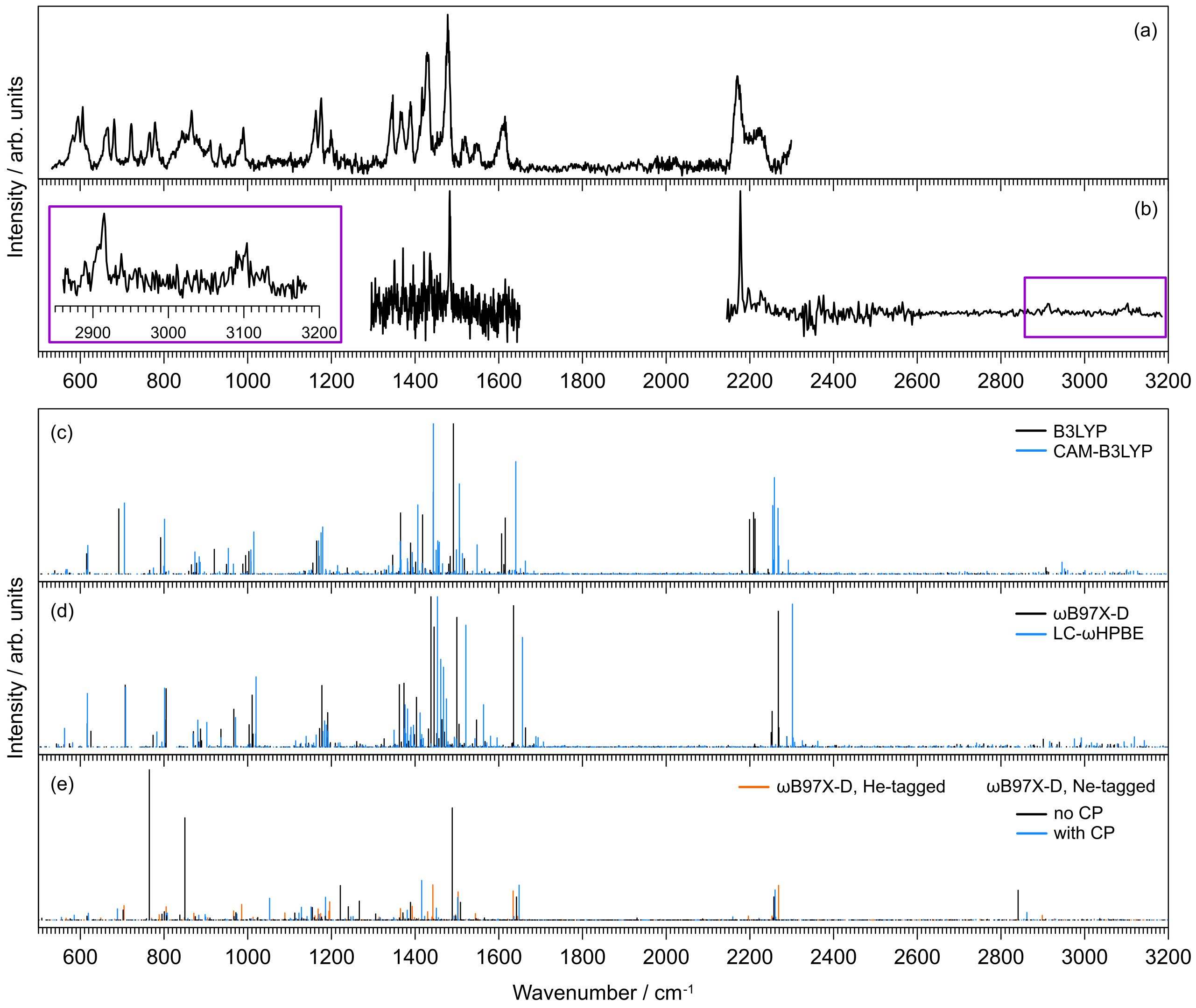

FIG. S3. IR spectroscopy of $2CNI^+$–Rg (Rg = He, Ne): (a) $2CNI^+$–Ne photodissociation spectrum recorded at FELIX. (b) $2CNI^+$–He photodissociation spectrum recorded in Edinburgh (the inset shows an expansion of the C-H stretching region). Because the beampath was not flushed for atmospheric contaminants, there is higher noise level in the He-tagged spectrum over the CO_2 asymmetric stretch region where the fluence from the OPO dropped. (c)–(d) Anharmonic spectra for untagged $2CNI^+$ computed using the B3LYP, CAM-B3LYP, ωB97X-D, and LC-ωHPBE functionals with the cc-pVTZ basis set. (e) Anharmonic spectra for tagged $2CNI^+$ computed at the ωB97X-D/cc-pVTZ level of theory. For Ne-tagging, anharmonic spectra were calculated with (blue) and without (black) counterpoise correction.

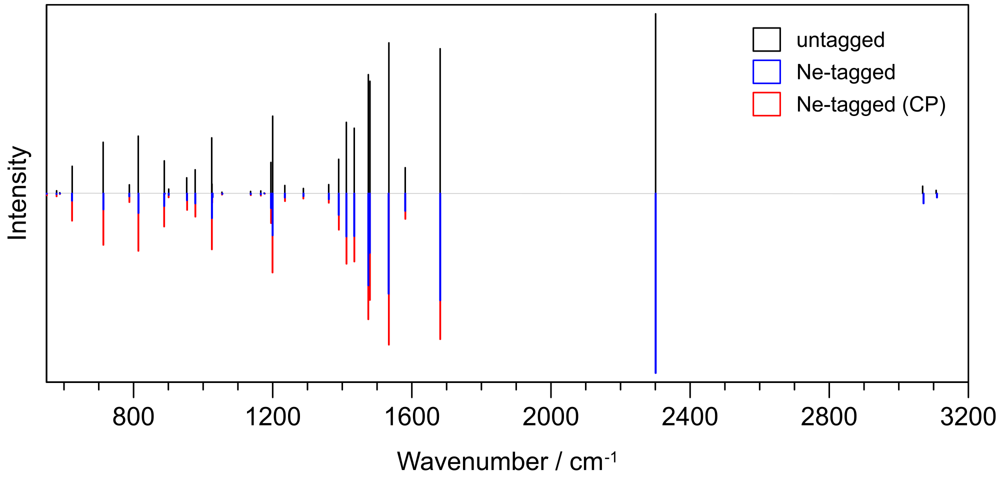

FIG. S4. Harmonic frequencies (computed at the ωB97X-D/cc-pVTZ level of theory) for the untagged (black) and Ne-tagged 2CNI$^+$. The Ne-tagged harmonic frequencies were computed with (red) and without (blue) counterpoise corrections. While there are some differences in intensity, there is little change in frequency. We note, however, that the experimental IR spectra reported in the manuscript have substantial intensity associated with combination bands and anharmonic couplings.

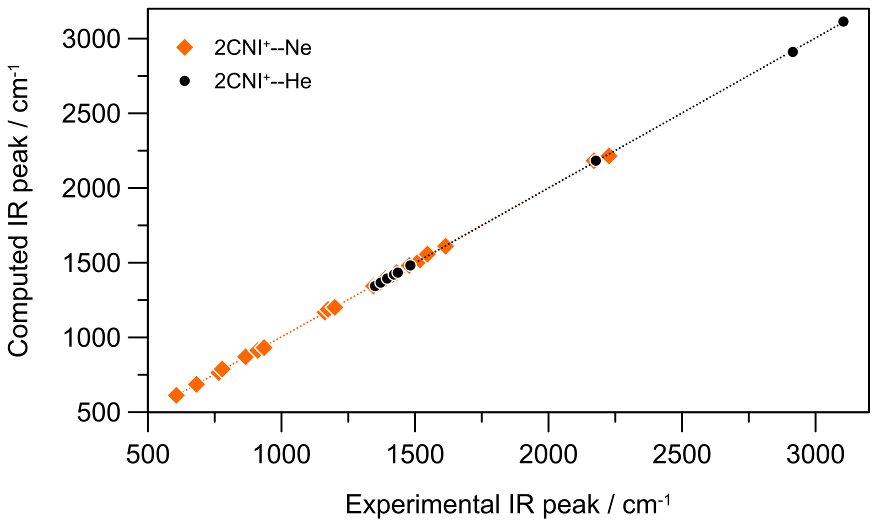

FIG. S5. Correlation between IR peak frequencies taken from the Ne-tagged (orange diamonds) and He-tagged (black circles) spectra for $2CNI^+$ and the B3LYP/N07D calculated frequencies. Mean absolute error (MAE) data is given in the following table.

TABLE S1. Mean absolute error (MAE) in cm^{-1} between the calculated anharmonic and experimental frequencies. The MAEs including the CN frequency are larger because computational methods apparently struggle to replicate the CN stretch frequency (change in dipole moment associated with the the CN stretch is large). Calculations used the cc-pVTZ basis set unless otherwise stated. CP indicates counterpoise corrected energies and gradients.

**Expt. tag**	**Method**	**MAE (exc. CN)**	**Average**	**MAE (inc. CN)**	**Average**
He	B3LYP	5.91	6.3	7.72	8.3
Ne	B3LYP	6.71		8.85	
He	CAM-B3LYP	16.40	15.4	28.63	24.2
Ne	CAM-B3LYP	14.30		19.68	
He	ωB97X-D	11.28	13.5	21.21	21.4
Ne	ωB97X-D	15.72		21.56	
He	LC-ωHPBE	23.64	26.1	48.86	46.1
Ne	LC-ωHPBE	28.57		43.24	
He	B3LYP/N07D	3.90	4.3	4.11	4.6
Ne	B3LYP/N07D	4.74		5.11	
He	ωB97X-D He-tag	14.23	-	33.51	-
Ne	ωB97X-D Ne-tag	32.93	-	50.90	-
Ne	ωB97X-D Ne-tag; CP	22.70	-	39.43	-

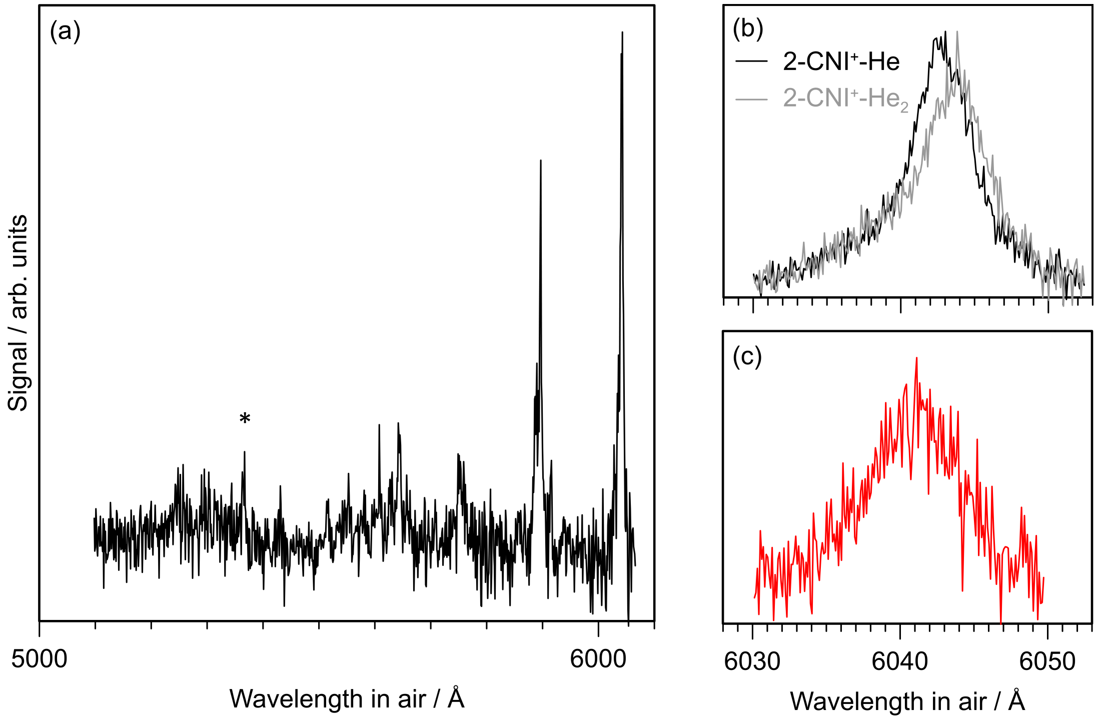

FIG. S6. Electronic spectroscopy of $2CNI^+$–He in terms of units of wavelength in air (Å): (a) Photodissociation spectrum for the $D_2 \leftarrow D_0$ transition. (b) Band origin measured for $2CNI^+$–He (black) and $2CNI^+$–He_2 (grey). (c) Two-colour measurement of the band origin. The feature denoted with the * in (a) was not reproduced in the simulation, and is either from anharmonic couplings (appears close in frequency to CN-stretching) not included in the simulation or a contaminant species in the experiment at the same m/z.

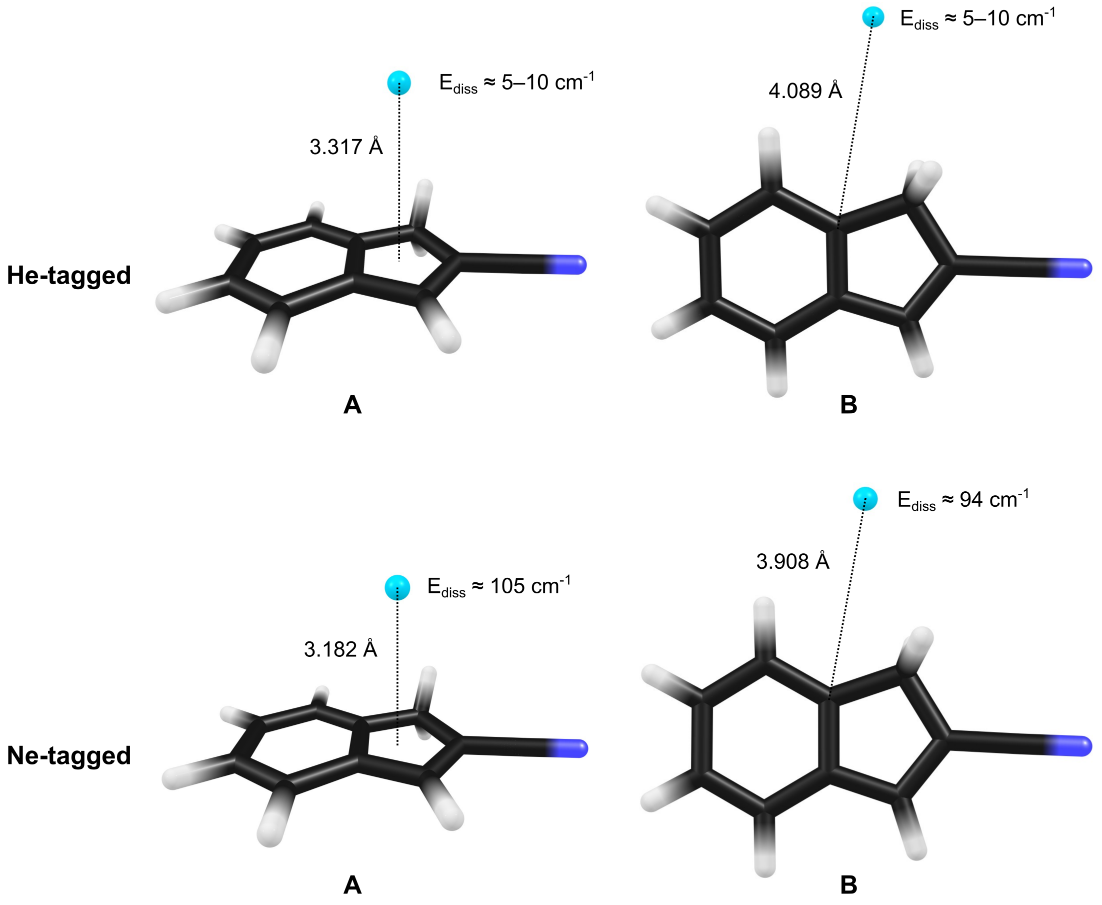

FIG. S7. Optimized structures for the lowest energy $2CNI^{+}$–Rg (Rg = He, Ne) species. Geometry A has the Rg atom above/below the molecular plane, while geometry B has the Rg atom in the molecular plane. At the CCSD(T)/cc-pVTZ level of theory (with ωB97X-D/cc-pVTZ zero-point energy corrections), geometry A is lowest in energy when counterpoise corrections are included, while B is lowest in energy when counterpoise corrections are excluded. However, the differences in relative energy are on the order of a few wavenumbers, which is similar to the expected prevision of the computational method. E_{diss} is the calculated tag atom dissociation energy.

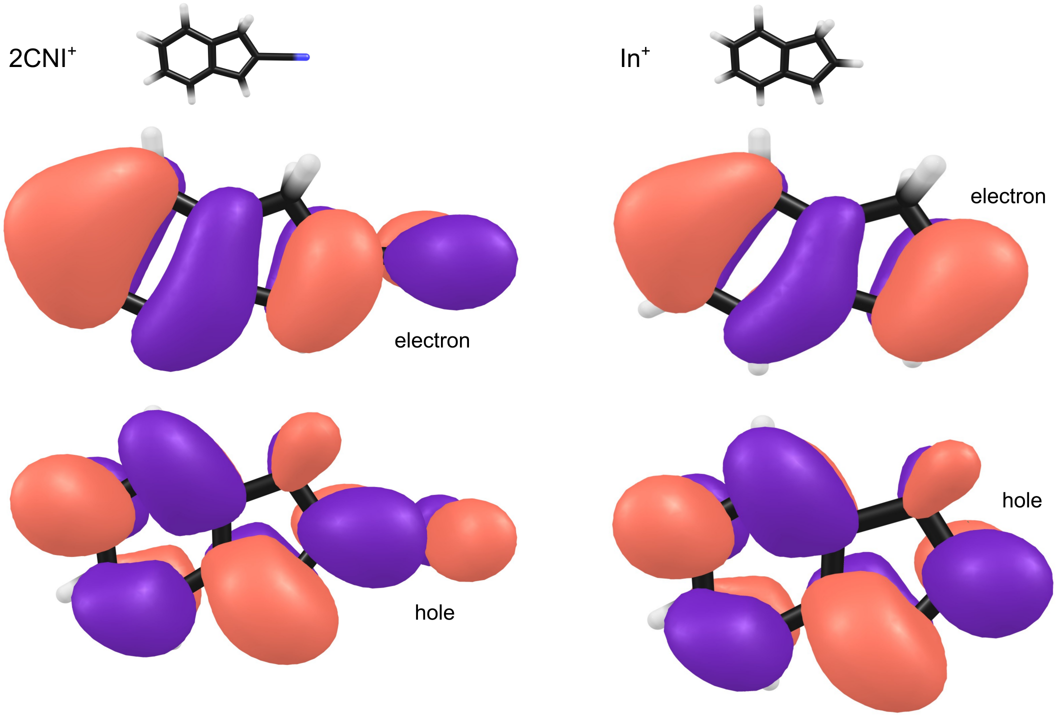

FIG. S8. Natural transition orbitals (NTOs) for the $D_2 \leftarrow D_0$ transition in $2CNI^+$ and the indene cation (In^+). Both transitions involve very similar molecular orbitals.

TABLE S2: Listing of $2CNI^+$ fundamental modes (B3LYP/N07D) and assignments.

Mode	Harmonic freq (cm^{-1})	Description
ν_1	3239.6	Aromatic CH stretch
ν_2	3230.1	Symmetric aromatic CH stretch
ν_3	3220.0	Symmetric aromatic CH stretch
ν_4	3216.5	Asymmetric aromatic CH stretch
ν_5	3208.1	Asymmetric aromatic CH stretch
ν_6	3080.6	Asymmetric aliphatic CH stretch
ν_7	3047.4	Asymmetric aliphatic CH stretch
ν_8	2251.6	CN stretch
ν_9	1653.3	CC skeletal stretch
ν_{10}	1553.1	CC skeletal stretch
ν_{11}	1512.4	CC skeletal stretch
ν_{12}	1465.6	CC skeletal stretch
ν_{13}	1452.7	CC skeletal stretch
ν_{14}	1426.1	CC skeletal stretch
ν_{15}	1403.9	CC skeletal stretch
ν_{16}	1369.6	CC skeletal stretch
ν_{17}	1340.4	CC skeletal stretch
ν_{18}	1267.3	CC skeletal stretch
ν_{19}	1223.6	Symmetric CH wag
ν_{20}	1185.7	Asymmetric in-plane aromatic CH bend
ν_{21}	1182.8	Asymmetric in-plane aromatic CH bend
ν_{22}	1158.7	Asymmetric aliphatic CH bend
ν_{23}	1157.9	Symmetric in-plane CH bend
ν_{24}	1124.0	Asymmetric in-plane CH bend
ν_{25}	1015.3	Asymmetric out-of-plane CH bend
ν_{26}	1013.2	Symmetric in-plane aromatic CH bend
ν_{27}	993.1	Asymmetric out-of-plane CH bend
ν_{28}	949.5	Aromatic (pent) CH wag
ν_{29}	933.1	Symmetric out-of-plane CH bend
ν_{30}	886.5	In-plane CC skeletal breathing
ν_{31}	877.1	In-plane CC skeletal breathing
ν_{32}	870.9	Asymmetric out-of-plane CH wag
ν_{33}	788.4	Symmetric out-of-plane aromatic CH wag
ν_{34}	777.5	In-plane CC skeletal breathing
ν_{35}	689.2	Asymmetric out-of-plane CH wag
ν_{36}	618.7	In-pane CC skeletal deformation
ν_{37}	582.1	In-pane CC skeletal deformation
ν_{38}	568.9	In-plane CCN bend
ν_{39}	542.2	Asymmetric out-of-plane CH wag (pent)
ν_{40}	466.2	Out-of-plane aromatic CH wag (benz)
ν_{41}	425.6	In-plane symmetric CC skeletal breathing
ν_{42}	413.9	Symmetric out-of-plane CH wag
ν_{43}	373.6	Asymmetric in-plane CC skeletal bend
ν_{44}	343.8	Symmetric out-of-plane CH wag
ν_{45}	226.1	Symmetric out-of-plane CH wag
ν_{46}	183.1	Out-of-plane CH wag
ν_{47}	132.9	In-plane CN wag
ν_{48}	77.6	Out-of-plane CN wag

TABLE S3. $2CNI^{+}$ geometry, optimized at the ωB97X-D/cc-pVTZ level of theory.

C	0.513772	0.739129	0.000008
C	0.492356	-0.693201	0.00001
C	1.691952	-1.448584	0.000005
C	2.873071	-0.765304	-0.000009
C	2.880662	0.647744	-0.000015
C	1.704836	1.408343	-0.000005
C	-0.838483	-1.131324	0.000008
H	1.660309	-2.528829	0.000009
H	3.814101	-1.295475	-0.000017
H	3.83391	1.159519	-0.000027
H	1.7543	2.487673	-0.000009
H	-1.171046	-2.158915	0.000004
C	-3.080809	-0.081831	-0.000004
C	-1.678246	-0.026479	0.00001
N	-4.233253	-0.111009	-0.000024
C	-0.89353	1.250538	0.000017
H	-1.126141	1.859443	0.877054
H	-1.126151	1.859461	-0.877003
